# ^18^F-FDG PET/CT in the Preoperative Diagnostic and Staging of Lung Cancer and as a Predictor of Lymph Node Involvement

**DOI:** 10.3390/jcm14041324

**Published:** 2025-02-17

**Authors:** Nathalie Viohl, Matthias Steinert, Martin Freesmeyer, Christian Kühnel, Robert Drescher

**Affiliations:** 1 Clinic of Nuclear Medicine, Jena University Hospital, 07743 Jena, Germany; nathalie.viohl@med.uni-jena.de (N.V.); christian.kuehnel@med.uni-jena.de (C.K.); robert.drescher@med.uni-jena.de (R.D.); 2 Clinic of Thoracic Surgery, Leipzig University Hospital, 04103 Leipzig, Germany; matthias.steinert@medizin.uni-leipzig.de

**Keywords:** lung cancer, thoracic surgery, PET/CT, FDG, lymph node staging

## Abstract

**Background/Objectives**: The aim of this study was to evaluate the efficacy and accuracy of PET imaging and performance in defining the preoperative TNM classification, especially the intrathoracic lymph node staging, of patients with lung cancer. **Methods**: A retrospective, single-institution study of consecutive patients with surgical therapy of lung cancer that were undergoing preoperative PET/CT scanning at the same center was conducted. A total of 104 patients were included. All patients underwent surgical evaluation with mediastinal and hilar lymph node sampling. Five patients with preoperative suspicion of N3 nodal status who were only tested for N2 were excluded from the observations and analyses of nodal status. **Results**: PET/CT staged the nodal status correctly in 85 out of 99 patients (85.9%); overstaging occurred in 7 patients (7.1%) and understaging in 7 patients (7.1%). The overall prevalence of lymph node metastases was 42.3%. When preoperative T classification was compared with postoperative histopathological T classification, 75% patients were correctly staged, 13.5% were overstaged, and 11.5% were understaged by PET/CT. In univariate analysis, lymph node involvement was significantly associated (*p* < 0.05) with the following primary tumor characteristics: increasing diameter (>35 mm), a maximum standardized uptake value > 9.5, and higher grading. The tumor diameter and the degree of differentiation were found to be factors influencing the SUV_max_ of the primary tumor as well. **Conclusions**: Our data show that integrated PET/CT provides high accuracy in the intrathoracic nodal staging and tumor expansion of lung cancer patients and emphasizes the continued need for surgical staging.

## 1. Introduction

Lung cancer represents the primary cause of cancer-related mortality in numerous countries [1]. For the curative treatment of patients without distant metastases, the local tumor extension and involvement of thoracic lymph nodes are the critical factors in the planning of the surgical strategy. Patients without a metastatic lymph node or with only ipsilateral intrapulmonary or hilar (N1) nodes are generally considered candidates for limited pulmonary resection. Patients with ipsilateral mediastinal lymph node involvement (N2) may be candidates for a combination of local and systemic treatment [2]. The involvement of contralateral mediastinal lymph nodes (N3) is a well-accepted criterion for inoperability. Therefore, it is of considerable clinical interest to evaluate the locoregional lymph nodes as accurately as possible in order to choose the optimal treatment modality [3,4].

Positron emission tomography (PET) combined with computer tomography (CT) represents a non-invasive imaging method for the visualization of functional and anatomic relations. This combination is now established in modern diagnostic imaging and allows oncological whole-body staging in less than 30 min [5].

International guidelines recommend whole-body FDG-PET/CT for the evaluation of suspected and the staging of proven lung cancer. The diagnostic benefit of FDG-PET/CT has been demonstrated in numerous studies and meta-analyses [6,7,8,9].

The aim of this study was to evaluate the accuracy of PET/CT imaging and performance in defining the preoperative TNM classification, to derive recommendations for reporting, and therefore to improve the reliability of PET/CT reporting in clinical practice.

## 2. Materials and Methods

### 2.1. Patient Population

**Figure 1:**
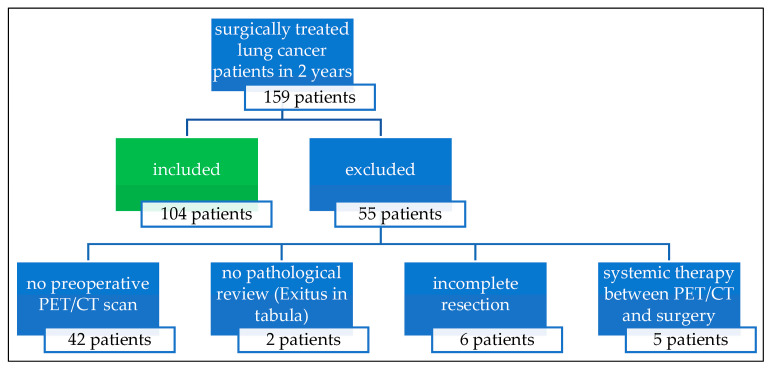
Patient selection: Of 159 patients who underwent surgery for lung cancer, 104 patients (65.4%) were included in the evaluation.

This study was conducted at a university hospital and tertiary referral center in Germany. Over a period of two years, thoracic surgery for the treatment of lung cancer was performed in 159 patients. A total of 104 patients (65.4%) who underwent a preoperative whole-body PET/CT to complete disease staging were included in the study and evaluated retrospectively (64 male, 40 female; median age 67.0 years, range 46–83 years). Patients who had PET/CT performed elsewhere, who received induction chemotherapy and/or radiation therapy, and patients with incomplete resection of the primary tumor or without histopathological processing of the specimen were excluded (Figure 1).

### 2.2. PET/CT Examinations

The median time interval between PET/CT and surgery was 18.5 days (range 1.0–113.0 days). In total, 67% of patients underwent PET/CT less than 4 weeks prior to surgery. Patients were positioned in the PET/CT scanner (Biograph mCT 40; Siemens Healthineers, Erlangen, Germany) in a supine position. All patients were fasting for at least 6 h prior. Blood glucose levels were below 11 mMol/L (median 5.9 mMol/L) before tracer injection. The intravenous injection of 3 MBq F-18 fluorodeoxyglucose (FDG) per kg body weight (median 268 MBq) was followed by a 60 to 90 min (median 71 min) uptake period. After the uptake period, a low-dose CT was acquired for attenuation correction and anatomical coregistration (3 mm slice thickness, 2 mm reconstruction interval, 100 kV, 40 mAs, CareDOSE4D). PET was acquired from the skull base to the upper thigh (2 min per bed position, reconstruction with TrueX/TOF, 3 iterations, 21 subsets, matrix 200 × 200 pixel, 5 mm Gaussian filter). To improve lung tissue delineation, an additional low-dose CT of the thorax in deep inspiration was added.

PET/CT studies were evaluated under the same standard by two nuclear medicine physicians (N.V., R.D.) with experience in CT imaging using the syngo.via software package (version VB60A_HF03; Siemens Healthineers, Erlangen, Germany). Glucose metabolism was determined by measuring maximum standardized uptake values (SUV_max_) with a volume-of-interest technique for lung masses, hilar and mediastinal lymph nodes, and extrathoracic findings suspicious for metastases. Mediastinal, blood pool, and hepatic values were measured for reference purposes. Lymph nodes were considered positive (suspicious for malignancy) if their tracer uptake was above the surrounding mediastinal background activity. For primary tumors and lymph nodes, long axis and short axis diameters, respectively, were measured on CT. Nodes were described as ipsi- or contralateral to the primary tumor. The image-based cTNM stage was determined according to the 8th edition of the IASLC TNM classification for lung cancer [10].

### 2.3. Surgical Treatment

Patients underwent thoracotomy, pulmonary resection, and systematic lymphadenectomy. Pulmonary resections included pneumonectomy (n = 14, 13.5%), bilobectomy (n = 7, 6.7%), lobectomy (n = 64, 61.5%), and segmentectomy (n = 19, 18.3%). Lymphadenectomy included removal of all accessible lymph nodes within the mediastinum and hilum. Intrapulmonary lymph nodes (levels 11 and 12) were excised from the resected lung specimen. A pathological review was conducted to evaluate the characteristics of the primary tumor and the status of lymph nodes. Immunohistochemistry was employed when deemed necessary. The histopathological pTNM stage was determined.

### 2.4. Statistical Analysis

PET/CT findings (positive/negative, SUV, lesion size) were correlated with histopathologic results (positive/negative, lesion size) for T, N, and M1a (intrathoracic metastases) variables. Univariate analyses for primary tumor characteristics and lymph node involvement associated with the SUV_max_ value and malignant involvement were performed on a per-patient basis using a chi-square test, Fischer’s exact test, unpaired *t*-test, and analysis of variance where appropriate. Statistical analyses were conducted using SPSS Statistics (IBM, Armonk, NY, USA) and Stata/IC (Stata Corporation, College Station, TX, USA). A *p* value of <0.05 was considered significant.

## 3. Results

### 3.1. Imaging and Histopathology Findings: T Category

**Figure 2:**
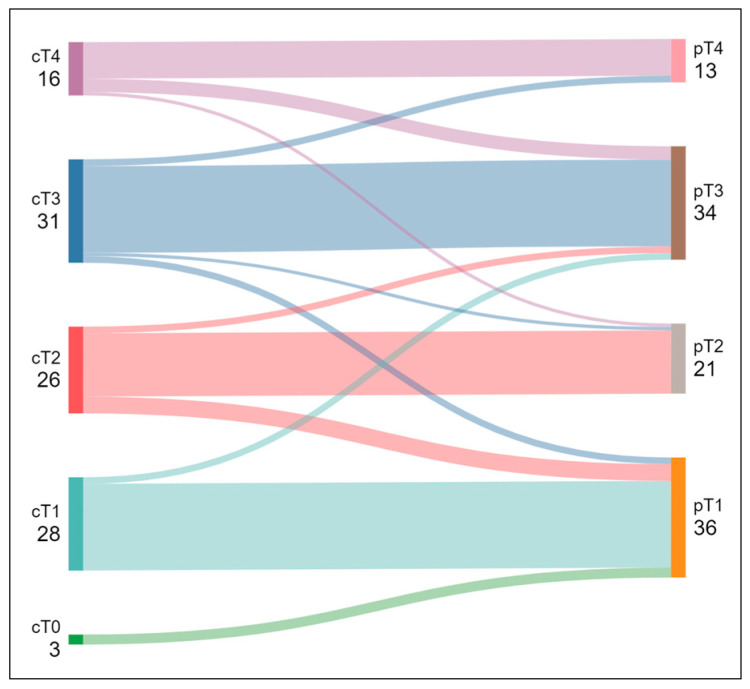
Changes in T category from preoperative PET/CT (left) to postoperative histopathology (right). In three cases, the lung lesion did not show glucose hypermetabolism on PET/CT (cT0). Subclassifications are not shown for clarity.

All PET/CT examinations were of diagnostic quality. The accuracy of the PET/CT for the T classification was 75%. In total, 13.5% were overstaged, and 11.5% were understaged (Figure 2, Table 1).

Median primary tumor size on imaging was 35 mm (range 8–170 mm), with a median SUV_max_ of 11.2 (range 1.2–38.1). A total of 75 tumors (72.1%) were localized in the upper or middle lobes, and 29 tumors (27.9%) were localized in the lower lobes.

Histopathology revealed 59 adenocarcinomas (56.8%), 33 squamous cell carcinomas (31.7%), 7 small-cell lung carcinomas (6.7%), and 5 other entities (4.8%; 2 NSCLC with neuroendocrine differentiation, 2 non-differentiated NSCLC, 1 mixed SCLC/squamous cell carcinoma). The grading of the primary tumor was G1 in 7 patients (6.7%), G2 in 45 patients (43.3%), and G3 in 44 patients (42.3%); in 8 patients (7.7%), the grading remained unclear.

### 3.2. Imaging and Histopathology Findings: N Category

PET/CT staged the nodal status correctly in 85 of 99 patients (85.9%); overstaging occurred in 7 patients (7.1%) and understaging in 7 patients (7.1%). In pN0 patients, the accuracy was 90% (54 patients); 6 patients were overstaged (3 to cN1, 3 to cN2). In pN1 patients, the accuracy was 61.5% (8 patients); 4 patients were understaged (to cN0), and 1 patient was overstaged (to cN2). In pN2 patients, the accuracy was 88.5% (23 patients); 3 patients were understaged (2 to cN0, 1 to cN1) (Table 2).

In our study population, the prevalence of lymph node metastases was 42.3%. Nodal status was as follows: pN0: 60 patients (57.7%), pN1: 13 patients (12.5%), pN2: 26 patients (25%), and pNX: 5 patients (4.8%).

### 3.3. Imaging and Histopathology Findings: M Category

PET/CT showed lesions suspicious for malignancy in the contralateral lung in five patients (cM1a), which were histopathologically confirmed. Regarding tumor lesions outside the thorax, a complete correlation between PET/CT and histopathologic findings was not possible because not all such suspicious lesions underwent histopathologic evaluation. On PET/CT, extrathoracic lesions were seen in 15 patients (14.4%), solitary in 6 patients (cM1b, stage IVA) and multiple in 9 patients (cM1c, stage IVB). Metastases were osseous, cerebral, hepatic, parotid, and adrenal. Eight of the nine lesions which underwent histopathology were malignant (88.9%); six were not examined.

### 3.4. Disease Stage Concordance Between PET/CT and Histopathology

**Figure 3:**
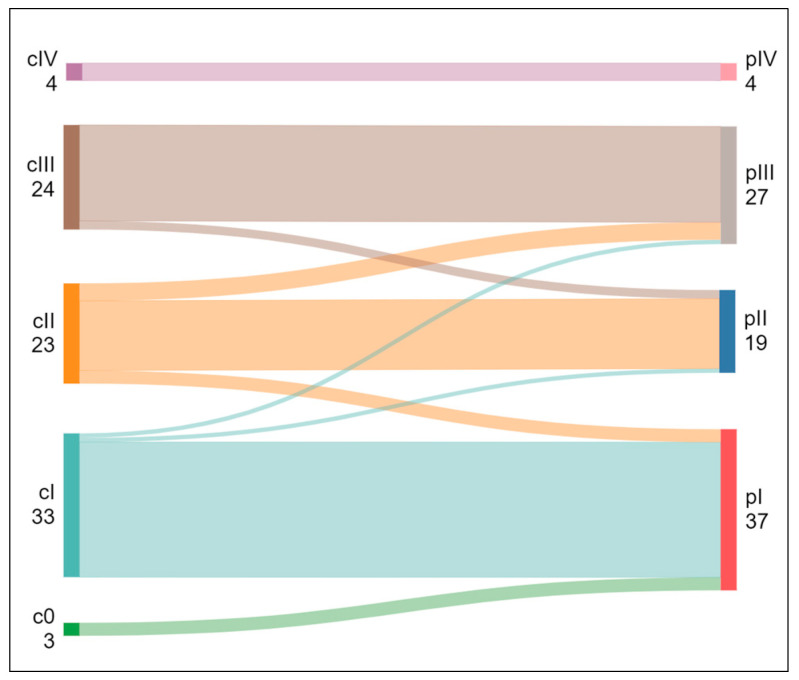
Changes in disease stage from preoperative PET/CT (c) to postoperative histopathology (p). Subclassifications are not shown for clarity.

In 68 of 87 patients (78.2%), the preoperatively assigned disease stage was confirmed histopathologically with regard to stage I/II/III/IV and subclassifications. Finally, there were 37 patients with stage I (3 IA1, 18 IA2, 9 IA3, and 7 IB), 19 patients with stage II (3 IIA and 16 IIB), 27 patients with stage III (17 IIIA and 10 IIIB), and 4 histologically proved patients with stage IVA (M1a). In 19 patients (21.8%), the disease stage was changed after surgery: 10 were understaged, and 9 were overstaged by PET/CT finding (Figure 3).

### 3.5. Factors Influencing Primary Tumor Glucose Metabolism

The size and the histopathologic grade of the primary tumor showed statistically significant correlations with tumor glucose metabolism measured on PET/CT.

**Figure 4:**
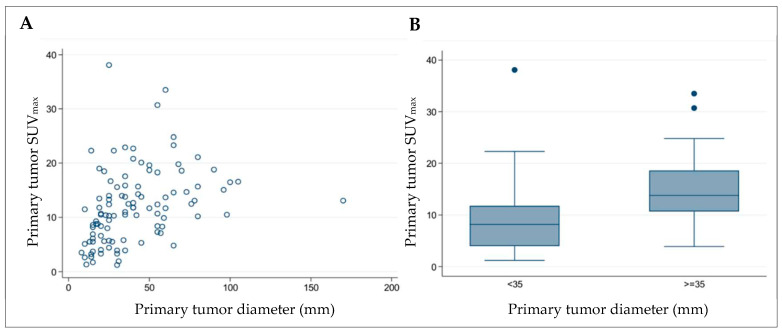
Correlation between primary tumor metabolism and diameter ((**A**) per patient and (**B**) categorized).

**Figure 5:**
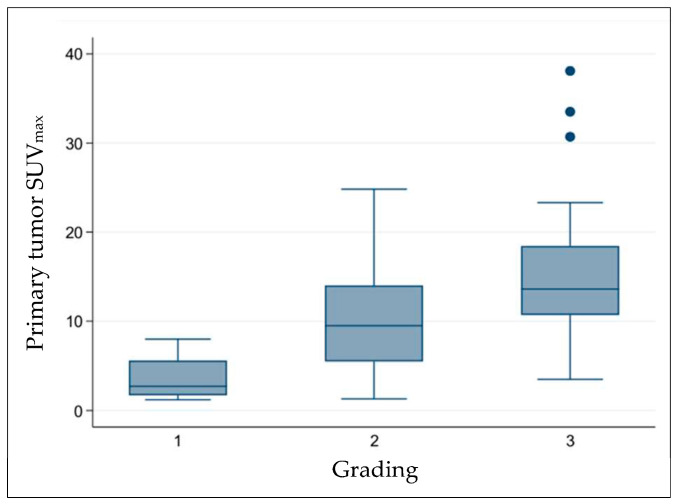
Correlation between primary tumor metabolism and histopathological grading.

- *Diameter of the primary tumor:* An increasing diameter of the primary tumor was statistically significantly associated with a higher SUV_max_ (*p* < 0.001; Figure 4A). Categorized into primary tumor diameters below and above 35 mm, SUV_max_ values were statistically significantly different (*p* < 0.001) (Figure 4B). For primary tumors below 35 mm (51 patients), the mean SUV_max_ was 9.1 ± 6.7; for those above 35 mm (53 patients), it was 10.6 ± 6.2.
- *Histopathologic grading of the primary tumor:* Tumor grading had a statistically significant influence on the SUV_max_ of the primary tumor. High-grade, less-differentiated lung cancers had a higher SUV_max_ than low-grade, well-differentiated lung cancers (*p* < 0.001). For G1, the mean SUV_max_ of the primary tumor was 3.5 ± 2.5; for G2, 10.6 ± 6.2, and for G3, 15.0 ± 6.9 (Figure 5).
- *Histopathologic tumor type:* No statistically significant influence of different tumor types on the glucose metabolism of the primary tumor could be determined in our patient cohort. Mean SUV_max_ was 13.1 ± 6.2 for squamous cell carcinoma, 11.3 ± 7.6 for adenocarcinoma, and 9.8 ± 5.5 for SCLC. The highest SUV_max_ was measured in patients with squamous cell and adenocarcinoma (33.5 and 38.1, respectively).

### 3.6. Factors Influencing Lymph Node Involvement

For the following correlation analyses, the patient cohort was divided into two groups: pN+: ‘histopathologic evidence of malignant lymph node(s) (including pN1, pN2)’ and pN0: ‘no histopathologic evidence of malignant lymph node(s)’.

- *SUV_max_ of the primary tumor:* SUV_max_ values of the primary tumor were categorized as 0–3, >3–5, >5–10, and >10, in line with the categorization used in clinical PET/CT reports in our institution. A higher SUV_max_ of the primary tumor was statistically significantly associated with lymph node involvement (Table 3). The mean SUV_max_ in pN0 and pN+ situations was 10.8 and 13.6, respectively (*p* = 0.026). The cut-point with a sensitivity of 79.5%, a specificity of 50%, a positive predictive value (PPV) of 53.8%, and a negative predictive value (NPV) of 76.9% was an SUV_max_ of the primary tumor of 9.5.

**Table 3:** Primary tumor SUV_max_ and lymph node involvement.

SUV_max_	Patients	pN0	pN+	*p* Value
0–3	6	6 (100%)	0 (0%)	
>3–5	10	9 (90%)	1 (10%)	
>5–10	25	15 (60%)	10 (40%)	
>10	63	30 (47.6%)	33 (52.4%)	0.016

- *Histopathologic grading of the primary tumor:* Higher tumor grading was a statistically significant risk factor for lymph node involvement (Table 4). In eight patients (7.7%), the histopathological grading remained unclear.

**Table 4:** Correlation of lymph node involvement and grading of the primary tumor.

Grading	Patients	pN0	pN+	*p* Value
1	7	7 (100%)	0 (0%)	
2	45	31 (68.9%)	14 (31.1%)	
3	44	18 (40.9%)	26 (59.1%)	0.001

- Diameter of the primary tumor: An increasing diameter of the primary tumor was statistically significantly associated with lymph node involvement (Table 5). The cut-point with a sensitivity of 63.6%, a specificity of 71.2%, a PPV of 62.2%, and an NPV of 72.4% was a diameter of the primary tumor of 37 mm.

**Table 5:** Correlation of lymph node involvement and diameter of the primary tumor.

	Patients	Diameter (mm)	*p* Value
pN0	59	34.4 ± 26.6	
pN+	44	48.8 ± 24.5	0.001

- Histopathologic tumor type: Lymph node metastases were more common in patients with SCLC (57.1%) than in those with adeno- or squamous cell carcinoma (40.7% and 36.4%, respectively), which was not statistically significant.

## 4. Discussion

The TNM classification system for lung cancer allows the determination of the stage of the disease and enables the differentiation between curative and non-curative therapeutic modalities. Therefore, accurate staging is of paramount importance [11]. The preoperative staging has been improved through the use of PET/CT compared to conventional CT, resulting in a significant reduction in unnecessary operations [12,13,14]. This is mainly due to more accurate lymph node staging and the detection of unexpected distant metastases [12,15].

The ^18^F-FDG-PET/CT has fundamental importance for staging primary lung cancers and is able to provide accurate clinical data in oncological therapy [16]. In the patient cohort analyzed in this study, the T classification was correctly staged in 75% of cases. There was an over- and underestimation of the T classification in 11.5% and 13.5% of cases, respectively. Reasons for false T classification were lymph nodes located adjacent to the tumor, peritumoral infiltrates or atelectasis, tumor progression during the time between PET/CT and resection, subjective measurements, as well as methodological reasons (for example, histopathological tumor treatment with solvents leading to a different measurement).

The N classification was correctly staged in 86% of cases. The values of our study are consistent with other studies in which the accuracy of the N classification in NSCLC was 65% and 81%, respectively [12,17]. There was an over- and underestimation of the N classification in 7.1% and 15% of cases, respectively. In comparison, other studies have reported an over- and underestimation of the N classification in 15% (5.7%) and 20% (13.8%) of the cases [12,17]. The 18F-FDG PET/CT TNM classification was in complete agreement with the postoperative histological TNM classification in 58.3% of patients. This compares to 43% in the literature [18]. In the statistical analyses, no statistically significant influencing factor could be identified with regard to concordance and discordance in T and N classifications. The factors examined were tumor type, grading, glucose metabolism (SUV_max_) of the primary tumor, and the time interval between preoperative PET/CT and surgery.

A number of predictive markers for the postoperative histological findings have already been identified. Specific primary tumor characteristics, including an SUV_max_ > 9, large diameter, central location, and vascular invasion, were found to be statistically significantly associated with the presence of lymph node metastasis at the time of diagnosis [17]. Another study identified T2+ stage and adenocarcinoma as additional risk factors for unexpected advanced lymph node involvement [19].

In this study, it could be confirmed that with an increasing SUV_max_ of the primary tumor, increasing tumor diameter, and poorer differentiation (higher grading), the rate of malignant lymph node involvement increases. It was demonstrated that lymph node metastases were statistically significantly more prevalent in cases with a higher primary tumor SUV_max_ (*p* = 0.026). The mean SUV_max_ of the pN0 group was 10.8, while that of the pN+ group was 13.6. A lymph node involvement was observed in 10% of cases with an SUV_max_ of the primary lesion below 5, in 40% of cases with an SUV_max_ of the primary lesion between 5 and 10, and in 52.4% of cases with an SUV_max_ of the primary lesion higher than 10. Similar results were observed by Miyasaka et al. [20], where 12.7% of patients presented with lymph node involvement when the SUV_max_ of the primary lesion was ≤10, and 41% of patients presented with lymph node involvement when the SUV_max_ of the primary lesion was >10. The calculated cut-point for the primary tumor was 9.5, with a sensitivity of 79.5%, a specificity of 50%, a positive predictive value (PPV) of 53.8%, and a negative predictive value (NPV) of 76.9%. This is in contrast to the findings of Endoh et al., who reported that the SUV_max_ of the primary tumor was not a significant factor in the occurrence of lymph node metastasis [21].

It could also be confirmed that an increasing diameter of the primary tumors was a risk factor for lymph node involvement (*p* = 0.001). The mean diameter was 34.4 mm in the pN0 group and 48.4 mm in the N+ group. The determined cut-point, at which the product’s sensitivity and specificity are at their maximum, yielded a maximum tumor diameter of 37 mm with a sensitivity of 63.6%, a specificity of 71.2%, a positive predictive value (PPV) of 62.2%, and a negative predictive value (NPV) of 72.4%. In clinical practice, however, the staging based on a fixed cut-point is considered unrealistic because multiple factors (including prior examinations, tumor size dynamics, clinical observations of the patient, laboratory values, etc.) must be taken into account when evaluating the preoperative PET/CT scan.

Furthermore, a poorer differentiation of the primary tumor (higher grading) was identified as a significant risk factor for lymph node involvement (*p* = 0.001). An N+ situation was observed in 0% of G1 carcinomas, 31% of G2 carcinomas, and 59% of G3 carcinomas. In eight patients, the grading remained unclear.

In contrast to another study, which found that adenocarcinomas exhibited a higher metastatic tendency than squamous cell carcinomas, the present study could not confirm an influence of the tumor type on lymph node involvement [22].

Another aim of this study was to evaluate if specific factors could be identified as a factor of influence on the SUV_max_ of the primary tumor. The SUV_max_ of the primary tumor showed a significant dependence on the tumor diameter. An increasing tumor diameter was associated with an increasing SUV_max_ of the primary tumor. With an SUV_max_ of the primary tumor of 0–3 (>3–5, >5–7, >7–10, >10), the average tumor diameter was 18.5 mm (26.2 mm, 21.9 mm, 33.5 mm, 49.4 mm). When dividing the collective into a maximum diameter of the primary tumor of less than or more than 35 mm, there was also proven a significant difference in the SUV_max_ of the primary tumor of both groups (mean value 9.1 and 14.7) (*p* < 0.001). Another study also demonstrated that the SUV_max_ was significantly higher (10.2) in cases with a tumor diameter > 3 cm compared to those with a diameter < 3 cm (5.6) [23].

Furthermore, carcinomas with poorer differentiation (higher grading) showed a significantly higher SUV_max_ than those with good differentiation (lower grading). The average SUV_max_ of the primary tumor was 3.5 for G1 tumors, 10.6 for G2 tumors, and 15.0 for G3 tumors.

There was no significant difference in the SUV_max_ of the primary tumor with regard to the different tumor types, while another study showed that squamous cell carcinomas had a significantly higher SUV_max_ than adenocarcinomas (6.4 vs. 4.6) [24].

Based on the present study and other studies, it can be concluded that ^18^F-FDG PET/CT should always be included in the preoperative diagnostic of patients with lung cancer, particularly if a curative treatment is intended, as it provides valuable information about the stage of the disease and for the patient-specific treatment strategy. A PET/CT-adjusted procedure can detect inoperable patients more reliably and can generally achieve a more efficient clinical procedure [25].

There were limitations to the present study. Not all extrathoracic lesions suspicious on PET/CT underwent histopathologic examination. Patient inclusion into the study focused on surgery; in a prospective follow-up study, all patients scheduled for PET/CT for lung cancer evaluation will be included. Although the SUV of FDG-PET was measured in a consisted manner, further standardization of PET/CT reporting is possible [26]. PET-positive mediastinal nodes were not systematically confirmed by biopsy before surgery. Lung cancer staging was conducted according to the 8th edition of the IASLC TNM classification for lung cancer. In the 9th edition, N2 and M1c stages are subdivided (N2a/N2b, single/multiple nodal metastases; M1c1/M1c2, metastases in one/in more than one extrathoracic organ system) [27]. The retrospective, single-center study only included a relatively small sample size, so the conclusions need to be verified by multicenter and larger sample size studies.

## 5. Conclusions

In this study, it could be confirmed that with an increasing SUV_max_ of the tumor, in-creasing tumor diameter, and poor differentiation (higher grading), lymph node involvement occurred significantly more often.

Tumor diameter and grading had an influence on the SUV_max_ of the primary tumor. Tumors with a larger diameter (>3.5 cm) and a higher grading showed a significantly higher SUV_max_ than smaller ones (<3.5 cm) and those with good differentiation (lower grading).

## Figures and Tables

**Table 1:** Preoperative PET/CT und postoperative histopathology: T category.

	n	pT1a	pT1b	pT1c	pT2a	pT2b	pT3	pT4
0	3	0 (0%)	3 (100%)	0 (0%)	0 (0%)	0 (0%)	0 (0%)	0 (0%)
cT1a	2	2 (100%)	0 (0%)	0 (0%)	0 (0%)	0 (0%)	0 (0%)	0 (0%)
cT1b	19	0 (0%)	16 (84.2%)	2 (10.5%)	0 (0%)	0 (0%)	1 (5.3%)	0 (0%)
cT1c	7	0 (0%)	0 (0%)	6 (85.7%)	0 (0%)	0 (0%)	1 (14.3%)	0 (0%)
cT2a	20	1 (5%)	1 (5%)	3 (15%)	12 (60%)	2 (10%)	1 (5%)	0 (0%)
cT2b	6	0 (0%)	0 (0%)	0 (0%)	0 (0%)	5 (83.3%)	1 (16.7%)	0 (0%)
cT3	31	0 (0%)	1 (3.2%)	1 (3.2%)	1 (3.2%)	0 (0%)	26 (83.9%)	2 (6.5%)
cT4	16	0 (0%)	0 (0%)	0 (0%)	0 (0%)	1 (6.2%)	4 (25.0%)	11 (68.8%)

**Table 2:** Preoperative PET/CT und postoperative histopathology: N category.

	Patients	pN0	pN1	pN2 *
Patients		60	13	26
cN0	60	54 (90.0%)	4 (6.7%)	2 (3.3%)
cN1	12	3 (23.1%)	8 (61.5%)	1 (7.7%)
cN2	27	3 (11.5%)	1 (3.8%)	23 (88.5%)

* Patients with preoperative PET/CT evidence of cN3 but only N1/N2 nodal surgery are not included.

## Data Availability

Due to data protection regulations, the data presented in this study are only available on request from the corresponding author.
